# A Rare Prose Poem

**Published:** 1917-02

**Authors:** 


### A Rare Prose Poem.

There was a quack doctor, who lived out in the hills who used two concoctions to cure human ills. The one was a physic, the other a drink; he made them of soda and colored them pink. One tweedledee and one tweedledum; the very same bottle was where they were from. When people were ailing they took them to bed, and swallowed whichever the quack doctor said. For people are easy and grafters are slick, and there are more suckers than fish in the creek. A quack politician came out in the hills; he turned the same trick for political ills. The one was protection, the other free trade; he had but one bottle from which they were made. They both skin you proper, and when it is done you can't tell to save you by which you were skun; for people are easy and grafters are slick, and there are more suckers than fish in the creek.—*Houston Chronicle.*

